# Structure-Based Virtual Screening: From Classical to Artificial Intelligence

**DOI:** 10.3389/fchem.2020.00343

**Published:** 2020-04-28

**Authors:** Eduardo Habib Bechelane Maia, Letícia Cristina Assis, Tiago Alves de Oliveira, Alisson Marques da Silva, Alex Gutterres Taranto

**Affiliations:** ^1^Laboratory of Pharmaceutical Medicinal Chemistry, Federal University of São João Del Rei, Divinópolis, Brazil; ^2^Federal Center for Technological Education of Minas Gerais—CEFET-MG, Belo Horizonte, Brazil

**Keywords:** SBVS, homology modeling, consensus virtual screening, scoring functions, computer-aided drug design

## Abstract

The drug development process is a major challenge in the pharmaceutical industry since it takes a substantial amount of time and money to move through all the phases of developing of a new drug. One extensively used method to minimize the cost and time for the drug development process is computer-aided drug design (CADD). CADD allows better focusing on experiments, which can reduce the time and cost involved in researching new drugs. In this context, structure-based virtual screening (SBVS) is robust and useful and is one of the most promising *in silico* techniques for drug design. SBVS attempts to predict the best interaction mode between two molecules to form a stable complex, and it uses scoring functions to estimate the force of non-covalent interactions between a ligand and molecular target. Thus, scoring functions are the main reason for the success or failure of SBVS software. Many software programs are used to perform SBVS, and since they use different algorithms, it is possible to obtain different results from different software using the same input. In the last decade, a new technique of SBVS called consensus virtual screening (CVS) has been used in some studies to increase the accuracy of SBVS and to reduce the false positives obtained in these experiments. An indispensable condition to be able to utilize SBVS is the availability of a 3D structure of the target protein. Some virtual databases, such as the Protein Data Bank, have been created to store the 3D structures of molecules. However, sometimes it is not possible to experimentally obtain the 3D structure. In this situation, the homology modeling methodology allows the prediction of the 3D structure of a protein from its amino acid sequence. This review presents an overview of the challenges involved in the use of CADD to perform SBVS, the areas where CADD tools support SBVS, a comparison between the most commonly used tools, and the techniques currently used in an attempt to reduce the time and cost in the drug development process. Finally, the final considerations demonstrate the importance of using SBVS in the drug development process.

## Introduction

In the past, the discovery of new drugs was made through random screening and empirical observations of the effects of natural products for known diseases.

**Figure 1:**
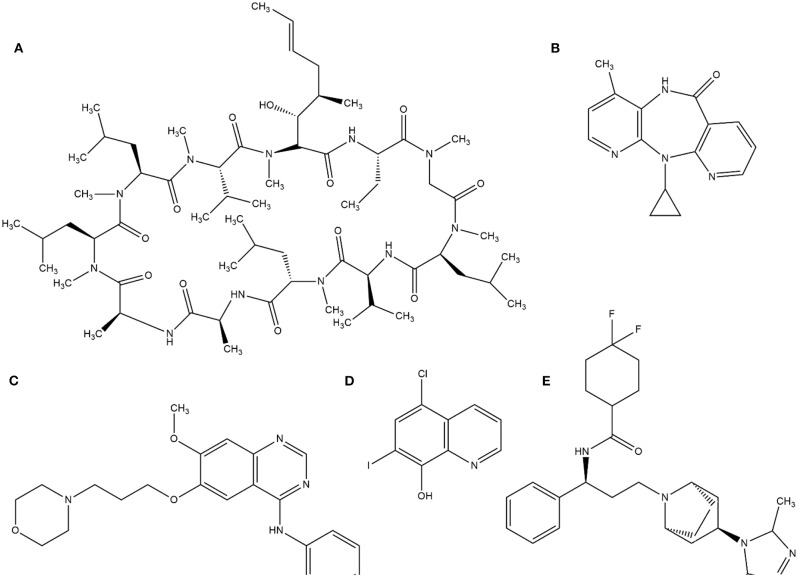
Examples of structures identified by HTS. **(A)** cyclosporine A, **(B)** Neviparine, **(C)** Gefitinib, **(D)** Clioquinol, and **(E)** Maraviroc.

This random screening process, although inefficient, led to the identification of several important compounds until the 1980s. Currently, this process is improved by high-throughput screening (HTS), which is suitable for automating the screening process of many thousands of compounds against a molecular target or cellular assay very quickly. The milestone of HTS was used in the identification of cyclosporine A as a immunosuppressant (von Wartburg and Traber, 1988). Subsequently, several drugs such as nevirapine (Merluzzi et al., 1990), gefitinib (Ward et al., 1994), and maraviroc (Wood and Armour, 2005) have reached the market. Notably, gefitinib was discovered by computational methods through a collection of 1500 compounds by ALLADIN (Martin, 1992) software. In addition, computational methods have been used to search successful compounds against malaria disease (Nunes et al., 2019). The structures of these molecules are in Figure 1.

Alternatively, the increased cost and evolution of medicines available in the last century have led to an improvement in the quality of life of the world population. However, while the average quality of life has been improved, a third of the population is still without access to essential medicines, which means that more than 2 billion people cannot afford to buy basic medicines (Leisinger et al., 2012). This problem is even worse in some places in Africa and Asia, where more than 50% of the people face problems obtaining medicines (Leisinger et al., 2012). Moreover, throughout the world, more than 18 million deaths that occur every year could be avoided, as well as tens of millions of deaths related to poverty and lack of access to essential medicines (Sridhar, 2008). The price of many medicines is inaccessible to limited-income populations and middle-income countries (Stevens and Huys, 2017).

While there is a need to increase the population's access to medicines, the pharmaceutical industry is facing unprecedented challenges in its business model (Paul et al., 2010). The current process of developing new drugs began to mature only in the second half of the twentieth century. The process evolved from observations made in the correlation of certain physical-chemical properties of organic molecules with biological potency. Optimization of these compounds by the incorporation of more favorable substituents resulted in more potent drugs. X-ray crystallography and nuclear magnetic resonance (NMR) techniques have provided information on the structures of enzymes and drug receptors. Many drugs, such as angiotensin-converting-enzyme (ACE) inhibitors, have been introduced to the clinical practice from this structural information.

**Figure 2:**
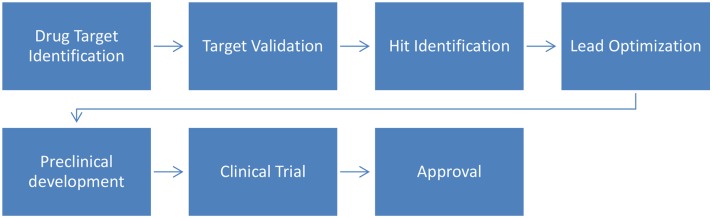
Drug development timeline.

The drug development process aims to identify bioactive compounds to assist in the treatment of diseases. In summary (Figure 2), the process starts with the identification of molecular targets for a given compound (natural or synthetic) and is followed by their validation. Then, virtual screening (VS) can be used to assist in hit identification (identification of active drug candidates) and lead optimization (biologically active compounds are transformed into appropriate drugs by improving their physicochemical properties). Finally these optimized leads will undergo preclinical and clinical trials to ultimately be approved by regulatory bodies (Lima et al., 2016).

In general, this process is time-consuming, laborious and expensive. The development of a new drug has an average cost between 1 and 2 billion USD and could take 10–17 years (Leelananda and Lindert, 2016), since it has to move through all phases for new drug development, from target discovery to drug registration. Even so, Arrowsmith (2012) showed that the probability of a drug candidate reaching the market after entering Phase I clinical trials fell from 10% in the 2002–2004 period to approximately 5% between 2006 and 2008, which represents a 50% decrease in just 4 years.

Thus, researchers are constantly investing in the development of new methods to increase the efficiency of the drug discovery process (Hillisch et al., 2004). The computer-aided drug design (CADD) approach, which employs molecular modeling techniques, has been used by researchers to increase the efficacy in the development of new drugs since it uses *in silico* simulations. Molecular modeling allows the analysis of many molecules in a short period of time, demonstrating how they interact with targets of pharmacological interest even before their synthesis. The technique allows the simulation and prediction of several essential factors, such as toxicity, activity, bioavailability and efficacy, even before the compound undergoes *in vitro* testing, thus allowing better planning and direction of the research (Ferreira et al., 2011). Better planning of the research means, in this case, fewer *in vitro* and *in vivo* experiments. Therefore, it reduces the run time and overall research costs.

**Figure 3:**
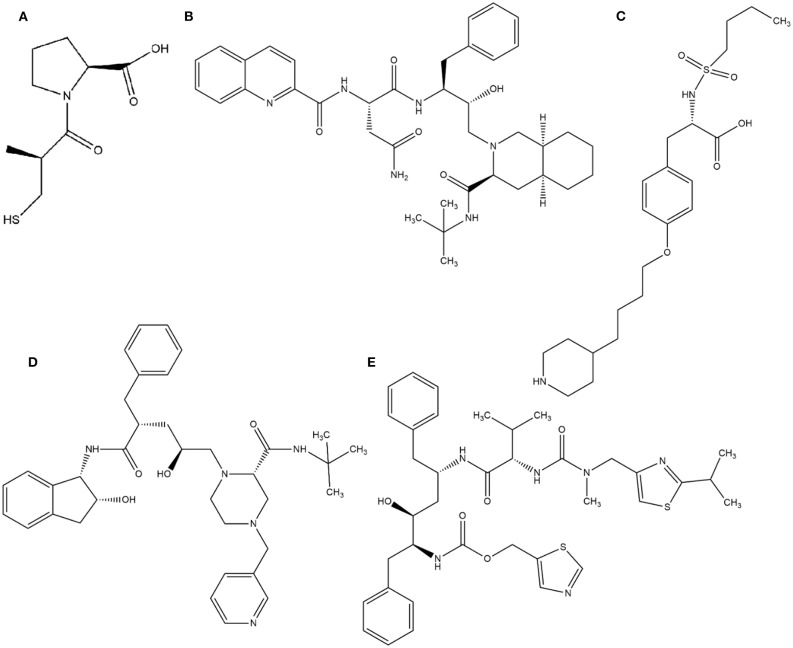
Drugs that came to the market with the assistance of VS: **(A)** Captopril, **(B)** Saquinavir, **(C)** Tirofiban, **(D)** Indinavir, **(E)** Ritonavir.

**Figure 4:**
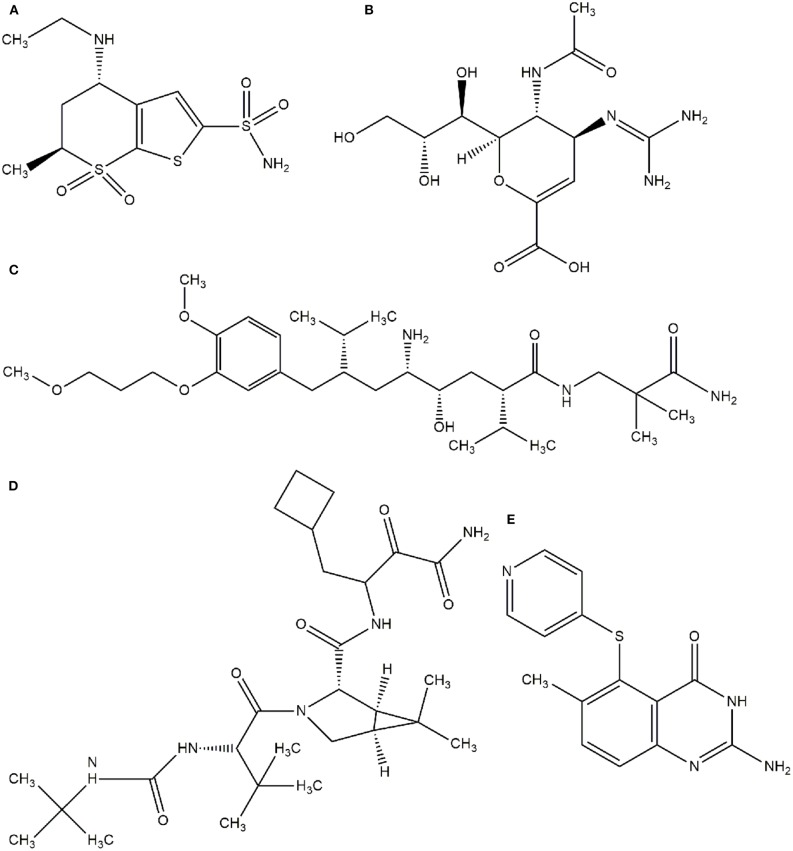
Drugs that came to the market with the assistance of VS. **(A)** Dorzolamide, **(B)** Zanamivir, **(C)** Aliskiren, **(D)** Boceprevir, **(E)** Nolatrexid.

In this context, virtual screening (VS) is a promising *in silico* technique used in the drug discovery process. An indispensable condition in performing virtual screening is the availability of a 3D structure of the target protein (Cavasotto, 2011). Therefore, some virtual databases were created to store 3D structures of molecules. Virtual screening is now widely applied in the development of new drugs and has already contributed to compounds on the market. Examples of drugs that came to the market with the assistance of VS include captopril (antihypertensive drug), saquinavir, ritonavir, and indinavir (three drugs for the treatment of human immunodeficiency virus), tirofiban (fibrinogen antagonist), dorzolamide (used to treat glaucoma), zanamivir (a selective antiviral for influenza virus), aliskiren (antihypertensive drug), boceprevir (protease inhibitor used for the treatment of hepatitis C), nolatrexed (in phase III clinical trial for the treatment of liver cancer) (Talele et al., 2010; Sliwoski et al., 2013; Devi et al., 2015; Nunes et al., 2019). The structures of these molecules are in Figures 3, 4.

This review will present an overview of the challenges involved in the development of new drugs. Section Computer-aided drug design (CADD) will describe CADD while section 3 will demonstrate how VS has been used as an agent in the process of developing of new drugs. Section Virtual screening (VS), in turn, will explain the main scoring functions used in recent scientific research. Section Consensus docking will explain consensus docking, which is a relatively unexplored topic in the virtual screening process. Section Virtual Databases will list the main virtual databases used in this task. Section Virtual screening algorithms presents the main VS algorithms used. Section Methods of evaluating the quality of a simulation will present some evaluation methods used to verify if the quality of the performed model/simulation is good. Section VS software programs, in turn, will present the main VS software currently used. Section Final considerations will present final considerations.

## Computer-Aided Drug Design (CADD)

One approach used to increase the effectiveness in the development of new drugs is the use of computer-aided drug design (CADD, well known as an *in silico* method) techniques, which uses a computational chemistry approach for the drug discovery process. CADD is a cyclic process for developing new drugs, in which all stages of design and analysis are performed by computer programs, operated by medicinal chemists (Oglic et al., 2018).

Strategies for CADD may vary, depending on what information about the target and ligand are available. In the early stage of the drug development process, it is normal for little or no information to exist about the target, ligands, or their structures. CADD techniques are able to obtain this information, such as which proteins can be targeted in pathogenesis and what are the possible active ligands that can inhibit these proteins. Kapetanovic (2008) briefly notes that CADD comprises (i) making the drug discovery and development process faster with the contribution of *in silico* simulations; (ii) optimizing and identifying new drugs using the computational approach to discover chemical and biological information about possible ligands and/or molecular targets; and (iii) using simulations to eliminate compounds with undesirable properties and selecting candidates with more chances for success. Recent software uses empirical molecular mechanics, quantum mechanics and, more recently, statistical mechanics. This last advancement allows the explicit effects of solvents to be incorporated (Das and Saha, 2017).

CADD gained prominence, as it allows obtaining information about the specific properties of a molecule, which can influence its interaction with the receptor. Thus, it has been considered a useful tool in rational planning and the discovery of new bioactive compounds. Alternatively, CADD simulations require a high computational cost, taking up to weeks if long jobs are used for molecular dynamics simulations. Therefore, it is a continuous challenge to find viable solutions that reduce the simulation runtime and simultaneously increase the accuracy of the simulations (Ripphausen et al., 2011). In this context, VS is a promising approach.

## Virtual Screening (Vs)

Popular VS techniques originated in the 1980s, but the first publication about VS appeared in 1997 (Horvath, 1997). In recent times, the use of VS techniques has been shown to be an excellent alternative to high throughput screening, especially in terms of cost-effectiveness and probability of finding the most appropriate result through a large virtual database (Surabhi and Singh, 2018).

**Figure 5:**
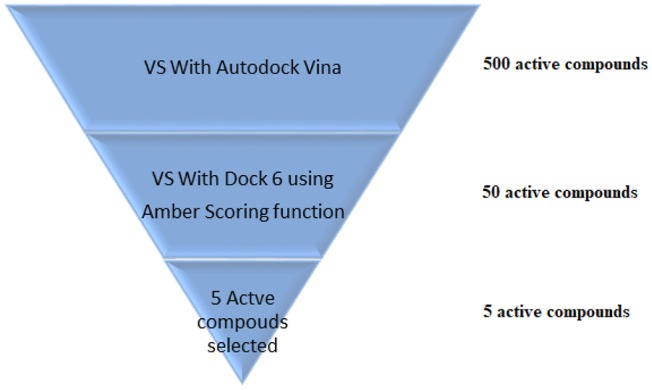
VS scheme.

VS is an *in silico* technique used in the drug discovery process. During VS, large databases of known 3D structures are automatically evaluated using computational methods (Maia et al., 2017). VS works like a funnel, by selecting more promising molecules for *in vitro* assays to be performed. In the example shown in Figure 5, it is assumed that a virtual screening will be performed on 500 possible active ligands for a target. Then, VS with AutoDock Vina (Trott and Olson, 2009) was carried out and the top 50 ligands were selected. Then, a VS using DOCK 6 (Allen et al., 2015) with the Amber scoring function was performed. DOCK 6 with Amber scoring function takes longer, because it performs molecular dynamics, but it promises better results. Finally, after VS with DOCK 6, the top 5 active compounds are selected to be purchased and then tested *in vitro*. With the use of VS, it is expected that those identified molecules are more susceptible to binding to the molecular target, which is typically a protein or enzyme receptor. Therefore, VS assists in identifying the most promising hits able to bind to the target protein or enzyme receptor, and only the most promising molecules are synthesized. In addition, VS identifies compounds that may be toxic or have unfavorable pharmacodynamic (for example, potency, affinity, selectivity) and pharmacokinetic (for example, absorption, metabolism, bioavailability) properties. Thus, VS techniques play a prominent role among strategies for the identification of new bioactive substances (Berman et al., 2013).

VS for drug discovery is becoming an essential tool to assist in fast and cost-effective lead discovery and drug optimization (Maia et al., 2017). This technique can aid in the discovery of bioactive molecules, since they allow the selection of compounds in a structure database that are most likely to show biological activity against a target of interest. After identification, these bioactive molecules undergo biological assays. In addition, there are VS techniques using machine learning methods that predict compounds with specific pharmacodynamic, pharmacokinetic or toxicological properties based on their structural and physicochemical properties that are derived from the ligand structure (Ma et al., 2009). Hence, VS tools play a prominent role among the strategies used for the identification of new bioactive substances, since they increase the speed of the drug discovery process as long as they automatically evaluate large compound libraries through computational simulations (Maithri and Narendra, 2016).

Structure based virtual screening (SBVS) is a robust, useful and promising *in silico* technique for drug design (Lionta et al., 2014). Therefore, this review will address SBVS, although there are other types of VS such as ligand-based virtual screening (Banegas-Luna et al., 2018) and fragment-based virtual screening (Wang et al., 2015).

### 4-Structure-Based Virtual Screening (SBVS)

Structure-based virtual screening (SBVS), also known as target-based virtual screening (TBVS), attempts to predict the best interaction between ligands against a molecular target to form a complex. As a result, the ligands are ranked according to their affinity to the target, and the most promising compounds are shown at the top of the list. SBVS methods require that the 3D structure of the target protein be known so that the interactions between the target and each chemical compound can be predicted *in silico* (Liu et al., 2018). In this strategy, the compounds are selected from a database and classified according to their affinity for the receptor site.

Among the techniques of SBVS, molecular docking is noteworthy due to its low computational cost and good results achieved (Meng et al., 2011). This technique emerged in the 1980s, when Kuntz et al. (1982) designed and tested a set of algorithms that could explore the geometrically feasible alignments of a ligand and target. However, although the approach was promising, it was only in the 1990s that it became widely used after there was an improvement in the techniques used in conjunction with an increase in the computational power and a greater access to the structural data of target molecules. During the execution of SBVS, the evaluated molecules are sorted according to their affinity to the receptor site. Hence, it is possible to identify ligands that are more likely to present some pharmacological activity with the molecular target. Score functions are used to verify the likelihood of a binding site describing the affinity between the ligand and target. In this process, a reliable scoring function is the critical component of the docking process (Leelananda and Lindert, 2016).

The use of SBVS has advantages and disadvantages. Among the advantages are the following:

- There is a decrease in the time and cost involved in the screening of millions of small molecules.
- There is no need for the physical existence of the molecule, so it can be tested computationally even before being synthesized.
- There are several tools available to assist SBVS.

The disadvantages can be highlighted as the following:

- Some tools work best in specific cases, but not in more general cases (Lionta et al., 2014).
- It is difficult to accurately predict the correct binding position and classification of compounds due to the difficulty of parameterizing the complexity of ligand-receptor binding interactions.
- It can generate false positives and false negatives.

Despite the disadvantages noted above, many studies using SBVS have been developed in recent years (Carregal et al., 2017; Mugumbate et al., 2017; Wójcikowski et al., 2017; Carpenter et al., 2018; Dutkiewicz and Mikstacka, 2018; Surabhi and Singh, 2018; Nunes et al., 2019), which shows that although SBVS has disadvantages, it is still wide used for developing drugs due to the reduction of time and cost. However, docking protocols are essential for achieving accurate SBVS. These protocols are composed of two main components: the search algorithm and the score function.

### Search Algorithms

Search algorithms are used to systematically search for ligand orientations and conformations at the binding site. A good docking protocol will achieve the most viable ligand conformations, in addition the most realistic position of the ligand at the binding site.

Thus, the search algorithm explores different positions of ligands at the active binding site using translational and rotational degrees of freedom in the case of rigid docking, while flexible docking adds conformational degrees of freedom to translations and rotations of the ligands. To predict the correct conformation of ligands, search algorithms adopt various techniques, such as checking the chemistry and geometry of the atoms involved [DOCK 6 (Allen et al., 2015), FLEXX (Rarey et al., 1996)], genetic algorithm [GOLD (Verdonk et al., 2003)] and incremental construction (Friesner et al., 2004). Algorithms that consider ligand flexibility can be divided into three types: systematic, stochastic and deterministic (Ruiz-Tagle et al., 2018). Some software uses more than one of these approaches to obtain better results.

Systematic search algorithms exploit the degrees of freedom of the molecules, usually through their incremental construction at the binding site. Increasing the degree of freedom (rotatable bonds) increases the number of evaluations needed to be performed by the algorithm. Increasing the degree of freedom (rotary links) increases the number of evaluations required to be performed by the algorithm, causing an increase in the time required for its execution. To reduce the time it takes to execute, termination criteria are inserted that prevent the algorithm from trying solutions that are in the space known to lead to wrong solutions. DOCK 6 (Allen et al., 2015), FLEXX (Rarey et al., 1996), and Glide (Friesner et al., 2004) are examples of software that uses systematic search algorithms.

Stochastic search algorithms perform random changes in the spatial conformation of the ligand, usually changing one system degree of freedom at a time, which leads to the exploration of several possible conformations (Ruiz-Tagle et al., 2018). The main problem of stochastic algorithms is the uncertainty of converging to a good solution. For this reason, to minimize this problem, several independent executions of stochastic algorithms are usually performed. Examples of stochastic research algorithms are Monte Carlo (MC) methods used by Glide (Friesner et al., 2004) and MOE (Vilar et al., 2008) and genetic algorithms used by GOLD (Verdonk et al., 2003) and AutoDock4 (Morris et al., 2009).

During the execution of a deterministic search algorithm, the initial state is responsible for determining the movement that can be made to generate the next state, which generally must be equal to or less in the energy from the initial state. One problem with deterministic algorithms is that they are often trapped in local minima because they cannot cross barriers; there are approaches, such as increasing the simulation temperature, that can be implemented to circumvent this problem. Energy minimization methods are an example of deterministic algorithms. Molecular dynamics (MD) is also an example of a deterministic search algorithm and is used by DOCK 6 (Allen et al., 2015). However, MD computational demands are very high, and while MD promises to have better results and ensures full-system flexibility, the runtime becomes a limiting factor for simulations because structure databases can have millions of ligands and targets.

### Scoring Functions

**Figure 6:**
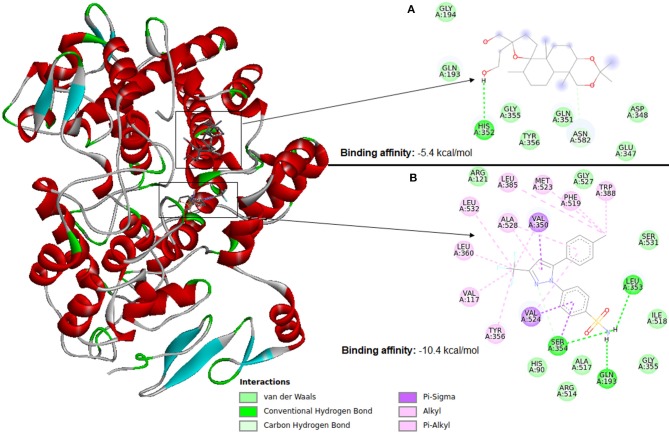
Identification of a ligand candidate by using a typical scoring function. The hydrogens were omitted for better visualization. **(A)** Inactive ligand, **(B)** celecoxib.

Molecular docking software uses scoring functions to estimate the force of non-covalent interactions between a ligand and molecular target using mathematical methods. A scoring function is one of the most important components in SBVS (Huang et al., 2010) as it is primarily responsible for predicting the binding affinity between a target and its ligand candidate. Thus, the scoring functions are the main reason for the success or failure of docking tools (ten Brink and Exner, 2009). Therefore, despite the wide use, the estimation of the interaction force between a ligand and molecular target remains a major challenge in VS. Figure 6 illustrates docking using Autodock Vina between cyclooxygenase-2 (PDB ID: 4PH9) and two ligands (a) an inactive ligand and (b) celecoxib (an anti-inflammatory). Compared to the inactive ligand, celecoxib is observed to have much more interactions with the protein, which causes celecoxib to form a more stable binding in the VS. This result causes the AutoDock Vina scoring function to see a binding energy of −10.4 kcal/mol for celecoxib and −5.4 kcal/mol for the inactive compound. The ligand with the highest binding affinity to the target can be selected for further testing. Therefore, in this case celecoxib would be chosen.

In general, there are three important applications of scoring functions in molecular docking. First, they can be used to determine the ligand binding site and the conformation between a target and ligand. This approach can be used to search for allosteric sites. Second, they can be used to predict the binding affinity between a protein and ligand. Third, they can also be used in lead optimization (Li et al., 2013).

Most authors define the scoring functions as three types (Huang et al., 2010; Ferreira et al., 2015; Haga et al., 2016): force field (FF), empirical and knowledge-based. Liu and Wang (2015) define two more types of scoring functions as: machine-learning-based and hybrid methods.

The force field scoring functions are based on the intermolecular interactions between the ligand and target atoms, such as the van der Waals, electrostatic and bond stretching/bending/torsional force interactions, obtained from experimental data and in accordance with the principles of molecular mechanics (Ferreira et al., 2015). Some published force-field scoring functions include the ones described in Li et al. (2015), Goldscore (Verdonk et al., 2003), and Sybyl/D-Score (Ash et al., 1997).

Empirical scoring functions estimate the binding free energy based on weighted structural parameters by adjusting the scoring functions to experimentally determine the binding constants of a set of complexes (Ferreira et al., 2015). To create an empirical scoring function, a set of data from protein-binding complexes whose affinities are known is initially used for training. A linear regression is then performed as a way of predicting the values of some variables (Huang et al., 2010). The weight constants generated by the empirical function are used as coefficients to adjust the equation terms. Each term of the function describes a type of physical event involved in the formation of the ligand-receptor complex. Thus, hydrogen bonding, ionic bonding, non-polar interactions, desolvation and entropic effects are considered. Some popular empirical, scoring functions include Glide-Score (Friesner et al., 2004), Sybyl-X/F-score (Certara, 2016) and DOCK 6 empirical force field (Allen et al., 2015).

In the knowledge-based scoring functions, the binding affinity is calculated by summing the binding interactions of the atoms of a protein and the molecular target (Ferreira et al., 2015). These functions consider statistical observations performed on large databases (Ferreira et al., 2015). The method uses pairwise energy potentials extracted from known ligand-receptor complexes to obtain a general scoring function. These methods assume that intermolecular interactions occurring near certain types of atoms or functional groups that occur more frequently are more likely to contribute favorably to the binding affinity. The final score is given as a sum of the score of all individual interactions. One example of software that uses a knowledge-based scoring function is ParaDockS (Meier et al., 2010).

In addition, machine-learning-based methods (Liu and Wang, 2015) have been considered as a fourth type of scoring function. Machine learning-based methods have gained attention for their reliable prediction (Pereira et al., 2016; Chen et al., 2018). Many researchers have used machine learning to improve SBVS algorithms, but we do not know any drugs developed after combining SBVS with machine learning. However, some researchers applied machine learning techniques to discover a new antibiotic capable of inhibiting the growth of *E. coli* bacteria (Stokes et al., 2020). These techniques have been used in quantitative structure-activity relationship (QSAR) analysis to predict various physical-chemical (for example, hydrophobicity, and stereochemistry of the molecule), biological (for example, activity and selectivity), and pharmaceutical (for example, absorption, and metabolism) properties of small molecule compounds. In these types of scoring functions, modern QSAR analyses can be applied to derive statistical models that calculate protein-ligand binding scores. Some scoring functions of this type are NNScore 2.0 (Durrant and McCammon, 2011), RF-Score-VS (Wójcikowski et al., 2017), SFCscoreRF (Zilian and Sotriffer, 2013), SVR-KB (Li et al., 2011), SVR-EP (Li et al., 2011), ID-Score (Li et al., 2013) and CScore (Ouyang et al., 2011).

There are some hybridized scoring functions that cannot easily be classified into any of the categories listed above because they combine two or more of the previously defined scoring function types [force field (FF), empirical, knowledge based and machine-learning-based] into one scoring function. Therefore, they are called hybrid scoring functions. In general, the hybrid scoring function is a linear combination of the two or more scoring function components derived from a multiple linear regression fitting procedure (Tanchuk et al., 2016). For example, the GalaxyDock score function is a hybrid of physics-based, empirical, and knowledge-based score terms that has the advantages of each component. As a result, the performance was improved in decoy pose discrimination tests (Baek et al., 2017). A few recently published examples of this type of scoring function include the hybrid scoring function developed by Tanchuk et al. (Tanchuk et al., 2016), which combines force field machine learning scoring functions; SMoG2016 (Geng et al., 2019), which combines knowledge-based and an empirical scoring functions; GalaxyDock BP2 (Baek et al., 2017), which combines force field, empirical, and knowledge-based scoring functions and iScore (Geng et al., 2019), which combines empirical and force-field scoring functions.

### Consensus Docking

In the last decade, a new technique of VS called consensus docking (CD) has been used in some studies (Park et al., 2014; Tuccinardi et al., 2014; Chermak et al., 2016; Poli et al., 2016; Aliebrahimi et al., 2017) to increase the accuracy of VS studies and to reduce the false positives obtained in VS experiments (Aliebrahimi et al., 2017).

This technique is a combination of two different approaches, in which the resultant combination is better than a single approach alone. However, Poli et al. (2016) reported that there are few studies that evaluate the possibility of combining the results from different VS methods to achieve higher success rates in VS studies.

Houston and Walkinshaw (2013) described the main reason for using this combination: the individual program may present incorrect results and these errors are mostly random. Therefore, even when two programs present different results, the combination of these results may, in principle, be much closer to the correct answer than even the best program alone. Houston and Walkinshaw also suggest that CD approaches using two different docking programs improve the precision of the predicted binding mode for any VS study. The same study also verified that a greater level of consensus in a given pose indicates a greater reliability in this result. Finally, the results presented by the authors suggest that the CD approach works as well as the best VS approaches available in the literature.

Park et al. (2014) use an approach in which they used a combination of the programs AutoDock 4.2 (Morris et al., 2009) and FlexX (Rarey et al., 1996) programs. These programs were chosen because both use different types of score functions (force field in AutoDock and empirical in FlexX). In this study, they achieved superior performance with the application of consensus docking than using each of the programs alone.

Alternatively, when using two different VS programs, there is extra time to run the two different tools and combine the results. However, Houston and Walkinshaw (2013) showed that the increased runtime may be advantageous; using AutoDock Vina (Trott and Olson, 2009) in a VS approach along with AutoDock4 (Morris et al., 2009) increased the final runtime by ~10%. This combination is interesting given the potential gains from its use.

Therefore, the use of consensus docking is a recent technique, and although there are few papers in the literature on the subject, it seems to be a promising approach for further VS studies.

## Virtual Databases

An indispensable condition in performing VS is the availability of a 3D structure of the target protein (Cavasotto, 2011) and ligands to be docked. Some databases were created to store 3D structures of molecules. Some of the free databases include Protein Data Bank (PDB) (Berman et al., 2013), PubChem (Kim et al., 2016), ChEMBL (Bento et al., 2014), ChemSpider (Pence and Williams, 2010), Zinc (Sterling and Irwin, 2015), Brazilian Malaria Molecular Targets (BraMMT) (Nunes et al., 2019), Drugbank (Wishart et al., 2018), and Our Own Molecular Targets (OOMT) (Carregal et al., 2013). In addition, there are some commercially available databases such as the MDL Drug Data Report^1^ Below we are going to present a brief explanation of each of these databases:

- Protein Data Bank (PDB) (Berman et al., 2013): PDB is the public database where three-dimensional structures of proteins, nucleic acids, and complex molecules have been deposited since 1971. The worldwide PDB organization ensures that PDB files are publicly available to the global community. It is widely used by the academic community and has grown consistently in recent years. In the last 10 years, the number of 3D structures of the PDB increased from 48,169 at the end of 2008 to 147,604 in the end of 2018, an increase of nearly 207%. This implies that in the last 10 years, almost 9,943 new structures have been added to the PDB every year, just over 27 structures per day, on average. The pace of this growth has increased. At the beginning of this decade approximately 25 new entries were added per day on average. In 2018, over 31 new structures were added per day, an average daily growth of 24% compared to 2010.
- PubChem (Kim et al., 2016): PubChem is a public database, aggregating information from smaller, more specific databases. It has more than 97 million compounds available.
- ChEMBL (Bento et al., 2014): ChEMBL is a database of bioactive molecules with medicinal properties maintained by the European Institute of Bioinformatics (EBI) of the European Molecular Biology Laboratory (EMBL). Currently, it has almost 2.3 million compounds and 15.2 million known biological activities.
- Zinc (Sterling and Irwin, 2015): Zinc is a free database of commercially available compounds for VS. Zinc has more than 230 million commercially available compounds in the 3D format. Zinc is maintained by Irwin and Shoichet Laboratories of the Department of Pharmaceutical Chemistry at the University of California, San Francisco (UCSF).
- NatProDB (Paixão and Pita, 2016): The State University of Feira de Santana has made NatProDB available. This database stores 3D structures of the semiarid biome. The pharmacological profile of compounds from the semiarid flora have not yet been studied, which has motivated our research group to deepen the research by their molecular targets (Taranto et al., 2015).
- Our Own Molecular Target (OOMT) (Carregal et al., 2013): OOMT is a special molecular target database because it has the biological assay for all its molecular targets, and includes specific targets for cancer, dengue, and malaria. OOMT was created by a group of researchers from Federal University of São João del-Rei (UFSJ).
- Brazilian Malaria Molecular Targets (BraMMT) (Nunes et al., 2019): The BRAMMT database comprises thirty-five molecular targets for *Plasmodium falciparum* retrieved from the PDB database. This database allows *in silico* virtual high throughput screening (vHTS) experiments against a pool of *P. falciparum* molecular targets.
- Drugbank (Wishart et al., 2018): DrugBank is a database that contains comprehensive molecular information about drugs, their mechanisms, their interactions, and their targets. The database contains more than 11,900 drug entries, including nearly 2,538 FDA-approved small molecule drugs, 1,670 biotechnology (protein / peptide) drugs approved by the FDA, 129 nutraceuticals and nearly 6,000 investigational drugs.

Commercially available Databases:

- MDL Drug Data Report (MDDR) (Sci Tegic Accelrys Inc, 2019): MDDR is a commercial database built from patent databases, publications and congresses. It has more than 260,000 biologically relevant compounds and approximately 10,000 compounds are added every year.
- ChemSpider (Pence and Williams, 2010): ChemSpider is a database of chemical substances owned by the Royal Society of Chemistry. It has more than 71 million chemical structures from over 250 data sources. ChemSpider allows downloading up to 1000 structures per day. Previous contact is needed for the download of more structures, and ChemSpider is therefore not a totally free database.

## Virtual Screening Algorithms

In VS, we are targeting proteins in the human body to find novel ligands that will bind to them. VS can be divided into two classes: structure-based and ligand-based. In structure-based virtual screening, a 3D structure of the target protein is known, and the goal is to identify ligands from a database of candidates that will have better affinity with the 3D structure of the target. VS can be performed using molecular docking, a computational process where ligands are moved in 3D space to find a configuration of the target and ligand that maximizes the scoring function. The ligands in the database are ranked according to their maximum score, and the best ones can be investigated further, e.g., by examining the mode and type of interaction that occurs. Additionally, VS techniques can be divided according to the algorithms used as follows:

- Machine Learning-based Algorithms
  - Artificial neural networks (ANNs) (Ashtawy and Mahapatra, 2018);
  - Support vector machines (Sengupta and Bandyopadhyay, 2014);
  - Bayesian techniques (Abdo et al., 2010);
  - Decision tree (Ho, 1998);
  - k-nearest neighbors (kNN) (Peterson et al., 2009);
  - Kohonen's SOMs and counterpropagation ANNs (Schneider et al., 2009);
  - Ensemble methods using machine learning (Korkmaz et al., 2015);
- Evolutionary Algorithms
  - Genetic algorithms (Xia et al., 2017);
  - Differential evolution (Friesner et al., 2004), Gold (Verdonk et al., 2003), Surflex (Spitzer and Jain, 2012) and FlexX (Hui-fang et al., 2010);
  - Ant colony optimization (Korb et al., 2009);
  - Tabu search (Baxter et al., 1998);
  - Particle swarm optimization (Gowthaman et al., 2015) and PSOVina (Ng et al., 2015);
- Local search such as Autodock Vina (Trott and Olson, 2009), SwissDock/EADock (Grosdidier et al., 2011) and GlamDock (Tietze and Apostolakis, 2007);
- Exhaustive search such as eHiTS (Zsoldos et al., 2007);
- Linear programming methods such as Simplex Method (Ruiz-Carmona et al., 2014);
- Systematic methods such as incremental construction used by FlexX (Rarey et al., 1996), Surflex (Spitzer and Jain, 2012), and Sybyl-X (Certara, 2016);
- Statistical methods
  - Monte Carlo (Harrison, 2010);
  - Simulated annealing (SA) (Doucet and Pelletier, 2007), Hatmal and Taha (Hatmal and Taha, 2017);
  - Conformational space annealing (CSA) (Shin et al., 2011);
- Similarity-based algorithms
  - Based on substructures (Tresadern et al., 2009);
  - Pharmacochemical (Cruz-Monteagudo et al., 2014);
  - Overlapping volumes (Leach et al., 2010);
  - Molecular interaction fields (MIFs) (Willett, 2006);
  - Hybrid approach (Morris et al., 2009; Haga et al., 2016);

After performing a VS simulation, it is necessary to verify whether the quality of the generated protein-ligand complexes can represent a complex that could be reproduced in experiments. There are several methods that can perform this assessment, which will be explained in the next section.

## Methods OF Evaluating The Quality of a Simulation

To verify the quality of a docking approach, some methods are used to evaluate generated complexes and to verify if the protein generated by the docking can reproduce the experimental data results of the ligand-receptor complex. The most common evaluation methods are root mean square deviation (RMSD) (Hawkins et al., 2008), receiver operating characteristic (ROC), area under the curve ROC (AUC-ROC) (Flach and Wu, 2005; Trott and Olson, 2009) enrichment factors (EFs) (Truchon and Bayly, 2007) and Boltzmann-enhanced discrimination of ROC (BEDROC) (Truchon and Bayly, 2007).

### Root-Mean-Square Deviation (RMSD)

One of the aspects evaluated in docking programs is the accuracy of the generated geometry (Jain, 2008). Docking programs attempt to reproduce the conformation of the ligand-receptor complex in a crystallographic structure. The metric root-mean-square deviation (RMSD) of atomic coordinates after the ideal superposition of rigid bodies of two structures is popular. Its popularity is because it allows the quantification of the differences between two structures, and these can be structures with the same and different amino acid sequences (Sargsyan et al., 2017). RMSD is widely used to evaluate the quality of a docking process performed by a program (Ding et al., 2016). The RMSD between two structures can be calculated according to the following equation (Sargsyan et al., 2017):

$$\begin{array}{l} RMSD\left(A,B\right)=\frac{1}{N}\overset{n}{\underset{i=1}{\sum }}d_{i}^{2}\; \end{array}$$

where d is the distance between atom i in the two structures and N is the total number of equivalent atoms. Since the calculation of RMSD requires the same number of atoms in both structures, it is often used in the calculation of only the heavy atoms or backbone of each amino acid residue.

Using the RMSD calculation, it is possible to evaluate if a program was able to reliably reproduce a known crystallographic conformation, as well as their respective intramolecular interactions. To verify if a given program can accomplish this task, ligand-targets complexes are subjected to a redocking process. After redocking, the overlap of the crystallographic ligand with the conformation of the ligand obtained with the docking program is then performed. Then, the RMSD calculation is used to check the average distance between the corresponding atoms (usually backbone atoms).

Generally, the RMSD threshold value is 2.0 Å (Jain, 2008; Meier et al., 2010; Gowthaman et al., 2015). However, for ligands with several dihedral angles, an RMSD value of 2.5 Å is considered acceptable (De Magalhães et al., 2004). In the case of binding a large ligand, some authors generally relax this criterion (Méndez et al., 2003; Verschueren et al., 2013). For a model generated by homology modeling, evaluating the RMSD value is important, although visual inspection of the generated model is also essential.

However, RMSD has some important limitations:

- RMSD can only compare structures with the same number of atoms;
- A small perturbation in just one part of the structure can create large RMSD values, suggesting that the two structures are very different, although they are not (Carugo, 2007);
- It has also been observed that RMSD values depend on the resolution of structures that are compared (Carugo, 2003);
- RMSD does not distinguish between a structure with some very rigid regions and some very flexible regions from a molecule in which all regions are semiflexible (Sargsyan et al., 2017);

Comparing the RMSD value of large structures may be significantly distorted from the commonly used 2Å threshold (Méndez et al., 2003). Despite these limitations, RMSD remains one of the most commonly used metrics to quantify differences between structures (Sargsyan et al., 2017).

**Figure 7:**
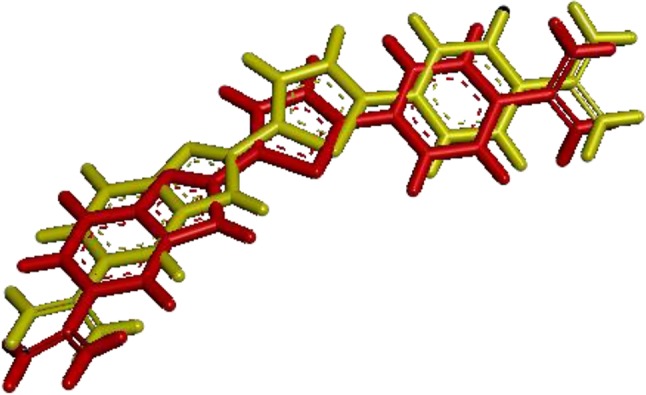
RMSD between the ligand FCP with a protein (PDB ID: 1VZK) after redocking using DOCK6.

Figure 7 shows the visualization of the FCP ligand superposed with its conformation after redocking to a protein (PDB ID: 1VZK, A Thiophene Based Diamidine Forms a “Super” AT Binding Minor Groove Agent). The RMSD between the crystallographic ligand and the same ligand after the redocking using DOCK6 is 0.97 Å. In the figure below, red represents the crystallographic ligand FCP and yellow represents FCP ligand after redocking using DOCK 6.

### ROC Curve and AUC

One of the great challenges of VS methods is the ability to differentiate true positive compounds (TPCs) against the target from false positive compounds (FPCs) (Awuni and Mu, 2015). Thus, it is important that VS tools have ways to assist their users in distinguishing TPCs from FPCs. The ROC curve and the area under the ROC curve (AUC-ROC) (Lätti et al., 2016) are widely used methodologies for this purpose.

TPC and decoys are used to create a ROC curve and AUC-ROC. TPCs are those with known biological activity for the molecular target of interest. Some databases, such as ChEMBL (Gaulton et al., 2012; Bento et al., 2014), allows users to search for these compounds. Alternatively, decoys are compounds that, although possessing physical properties similar to a TPC (such as molecular mass, number of rotatable bonds, and logP), have different chemical structures that make them inactive. They are generated from random molecular modifications in the structure of a TPC (Huang et al., 2006). Some databases, such as DUD-E (Mysinger et al., 2012) and Zinc (Sterling and Irwin, 2015), provide decoys for compounds of interest. DUD-E generates 50 different decoys for each TPC. The idea of using DUD-E decoys in VS is that the result of VS is more reliable if the program can separate TPCs from FPCs generated by DUD-E because FPCs have many TPC-like physical properties but are known to be inactive. A small number (>2) of known TPCs have to be used to calculate an AUC-ROC (Lätti et al., 2016).

After generating decoys, a VS process is performed using known TPCs and decoys against a target of interest (Yuriev and Ramsland, 2013). For each ligand-target complex, an affinity energy is then calculated. TPCs are expected to have lower affinity energy than inactive compounds. The ROC curve plots the distribution of true and false results on a graph, while AUC-ROC allows the evaluation of the probability of a result to be false. Hence, AUC-ROC reflects the probability of recovering an active compound preferentially to inactive compounds (Triballeau et al., 2005; Zhao et al., 2009), allowing verification of the sensitivity of a VS experiment in relation to its specificity. The larger the area under the curve, the better the ability to have a TPC and fewer FPC.

The AUC value can vary between 0 and 1. Hamza (Hamza et al., 2012) showed a practical way of interpreting the AUC values:

- AUC between 0.90 and 1.00: Excellent
- AUC between 0.80 and 0.90: Good
- AUC between 0.70 and 0.80: Fair
- AUC between 0.60 and 0.70: Poor
- AUC between 0.50 and 0.60: Failure

Therefore, the closer the AUC is to 1, the greater the ability of the VS tool to separate between TPCs and FPCs. AUC-ROC values close to 0.5 indicate a random process (Ogrizek et al., 2015). Acceptable values should be >0.7.

**Figure 8:**
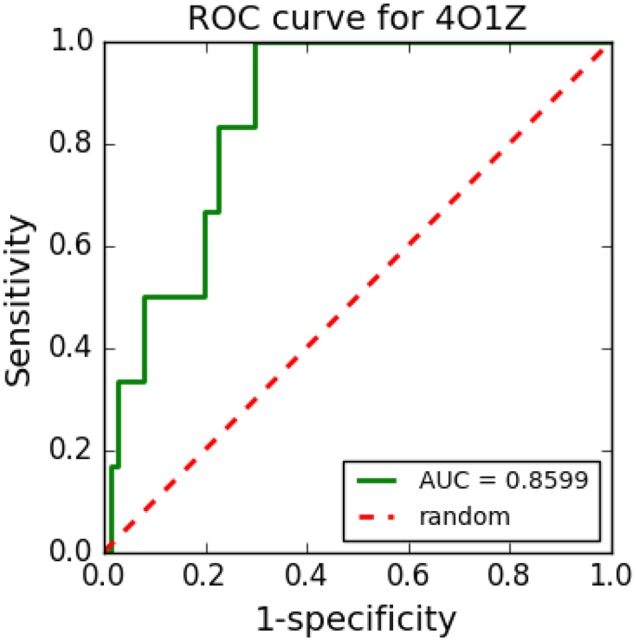
ROC curve example.

Figure 8 shows an example of an ROC curve generated in a VS performed with cyclooxygenase-1 complexed with meloxicam (PDB ID: 4O1Z) protein using five TPCs and 250 decoys. The VS tool was able to distinguish well between TPCs and FPCs with the generated ROC curve and its respective AUC, which was 0.8628.

### Boltzmann-Enhanced Discrimination of ROC (BEDROC)

There is much criticism in the use of the ROC curve as a method to measure virtual screening performance because it does not highlight the best ranked active compounds that would be used in *in vitro* experiments, which is called early recognition. Thus, Tuchon and Bayly (Truchon and Bayly, 2007) proposed Boltzmann-Enhanced Discrimination of ROC (BEDROC), which uses exponential weighting to give early rankings of active compounds more weight than late rankings of active compounds. However, Nicholls (Nicholls, 2008) say that AUC-ROC and BEDROC correlate when considering virtual screening simulations, and therefore, the ROC curve is a sufficient metric for performance measurements.

### Enrichment Factors (EFs)

The enrichment factor (EF) consists of the number of active compounds found in a fraction of 0 < χ <1 in relation to the number of active compounds that would be found after a random search (Truchon and Bayly, 2007). EFs are often calculated against a given percentage of the database. For example, EF10% represents the value obtained when 10% of the database is screened. EFs can be defined by the following formula (1):

(1) $$EF=\frac{\sum_{1}^{n}\delta_{i}}{\chi^{n}}\text{ where }\delta_{i}=\{\begin{array}{c} 1,\;r_{i}\;\leq \;\chi N\;\\ 0,\;r_{i}>\;\chi N \end{array}$$

r_i_ is the rank of the *i*th active compound in the list, N is the total number of compounds and n is the number corresponding to the selected compounds. The maximum value of EF is 1 / χ if x ≥ n / N and N / n if χ < n / N. The minimum value for EF is 0.

EF is quite simple, but it has some disadvantages. The EF, in addition to depending on the value set for χ, depends on the number of true positives and true negatives, which makes it another measure of experiment performance rather than measuring method performance (Nicholls, 2011). Another disadvantage of EF is that it weighs active compounds equally within the cutoff, so it is not possible to distinguish the best ranking algorithm in which all active compounds are ranked at the beginning of the ordered list of a worse algorithm and they are sorted immediately before the cutoff value [saturation effect (Lopes et al., 2017)].

The relative enrichment factor (REF) proposed by von Korff et al. (2009) eliminates the problem associated with the saturation effect by normalizing the EF by the maximum possible enrichment. Consequently, REF has well-defined boundaries and is less subject to the saturation effect.

## Vs Software Programs

There are several VS software programs using different docking algorithms that make a VS process easier for the researchers to execute by avoiding the need to have advanced knowledge of computer science and on how to implement the algorithms used in this task. In this regard, VS software can act as a possible cost reducer, since they function as filters that select from a database with thousands of molecules that are more likely to present biological activity against a target of interest. VS programs measure the affinity energy of a small molecule (ligand) to a molecular target of interest to determine the interaction energy of the resulting complex (Carregal et al., 2017).

Table 1 summarizes the main characteristics of the most used software in VS. The first column contains the software used and its reference. The second column contains the type of software license: free for academic use, freeware, open-source, or commercial. The free for academic use license indicates that the software in question can be used for teaching and research in the academic world without a fee. However, it implies that the software has restrictions for commercial use. A freeware license indicates that the software is free. Thus, users can use it without a fee, and all the functions of the program are available to be used without any restrictions. An open-source license indicates that the software source code is accessible so users can study, change, and distribute the software to anyone and for any purpose. Software developed under a commercial license indicates that it is designed and developed for a commercial purpose. Thus, in general, it is necessary to pay some licensing fee for its use. The third column indicates on which platforms the software can be used (Windows, Linux, or Mac). The next column indicates whether or not the software may consider protein flexibility during anchoring. The docking algorithm column lists the algorithms used by the software to perform the docking. The sixth column, called the scoring function, indicates which scoring functions are used by the software.

**Table 1:** Virtual screening software.

**Software**	**License**	**Platform**	**Protein flexibility**	**Docking algorithm**	**Scoring function**
AutoDock4 (Morris et al., 2009)	Free for academic use	Windows, Linux and Mac	Yes	Genetic algorithm Simulated annealing	Hybrid (Force-field and empirical)
Autodock Vina (Trott and Olson, 2009)	Open- source	Windows, Linux and Mac	Yes	Genetic algorithm Simulated annealing Local search Particle swarm optimization	Hybrid (Empirical and knowledge-based)
DOCK 6 (Allen et al., 2015)	Free for academic use	Windows, Linux and Mac	Yes	Shape fitting (sphere sets) Lowest energy binding	Force-Field Empirical
SwissDock/EADock DSS (Grosdidier et al., 2011)	Free for academic use	Web	No	Stochastic (Tabu search based) Local search Combination of broad and local search of the conformational space	Force-field
eHiTS (Zsoldos et al., 2007)	Freeware for academic use	Unix	No	Exhaustive search	Hybrid (Empirical and knowledge-based)
FITTED (Corbeil et al., 2007, 2008)	Commercial	Linux, Windows and Mac	Yes	Genetic algorithm	Force-field
FlexX (Rarey et al., 1996)	Commercial	Windows and Linux	No	Incremental construction	Empirical
FLIPDock (Zhao and Sanner, 2007)	Freeware for academic Use	Linux e Windows	Yes	Genetic algorithm	Force-field
Fred (McGann, 2011)	Free for academic use	Windows, Linux and Mac	No	Exhaustive search algorithm	Hybrid
GalaxyDock2 (Shin et al., 2013)	Freeware	Linux	Yes	Conformational analysis Genetic algorithm	Force-field
GeauxDock (Fang et al., 2016)	Open-source	Linux	Yes	Monte Carlo	Hybrid (Empirical and knowledge-based)
GlamDock (Tietze and Apostolakis, 2007)	Freeware	Windows, Linux and Mac	No	Monte Carlo Simulated annealing Local search Conformational analysis	Empirical
Glide (Friesner et al., 2004)	Commercial	Windows, Linux	Yes	Conformational analysis Monte Carlo sampling	Empirical
GOLD (Verdonk et al., 2003)	Commercial	Linux and Windows	Yes	Genetic algorithm	Force-field
ICM (Abagyan et al., 1994)	Commercial	Windows, Linux and Mac	Yes	Monte Carlo minimization	Force-field
iGEMDOCK/GEMDOCK (Hsu et al., 2011)	Freeware	Windows and Linux	Yes	Genetic algorithm	Empirical
LigandFit (Montes et al., 2007)	Commercial	Linux	Yes	Monte Carlo	Force-field
LigDockCSA (Shin et al., 2011)	–	–	Yes	Conformational space annealing Global optimization	Hybrid (Empirical and Force-field)
MOE (Vilar et al., 2008)	Commercial	Windows, Linux and Mac	Yes	Conformational analysis	Empirical, Force-field
ParaDockS (Meier et al., 2010)	Freeware	Linux	No	Genetic algorithm	Hybrid (Knowledge-based and empirical)
rDOCK (Ruiz-Carmona et al., 2014)	Open-source	Linux	Yes	Genetic algorithm, Monte CarloSimplex minimization	Hybrid (Empirical and force-field)
SLIDE (Schnecke and Kuhn, 2000)	Free for academic use	Linux	Yes	Conformational analysis	Empirical
Surflex (Spitzer and Jain, 2012)	Commercial	Windows, Linux and Mac	Yes	Incremental xonstruction	Empirical
Sybyl-X (Certara, 2016)	Commercial	Windows	Yes	Incremental construction	Force field
vLifeDock (Chopade, 2015)	Commercial	Windows, Linux and Mac	Yes	Genetic algorithm	Empirical

## Final Considerations

CADD has been used to improve the drug development process. In the past, the discovery of new drugs was often conducted through the empirical observation of the effect of natural products in known diseases. Thus, several possible drug candidates were tested without efficacy, and thereby wasted resources. The use of CADD allows for improving the development of new biologically active compounds and decreasing the time and cost for the development of a new drug. Thus, the emergence of SBVS has improved the drug discovery process and was established as one of the most promising *in silico* techniques for drug design.

This review verified that CADD approaches can contribute to many stages of the drug discovery process, notably to perform a search for active compounds by VS.

The use of techniques, such as SBVS, has limitations, such as the possibility of generating false positives and correct ranking of ligands docked. Moreover, there are several CADD methods and it is possible to obtain different results for the same input in different software. However, reducing the time and cost of the new drug development process as well as the constant improvement of existing docking tools indicates that CADD techniques will be one of the most promising techniques in the drug discovery process over the next years.

In the last decade, many studies have applied artificial intelligence in CADD to obtain more accurate models. Thus, most studies and future innovations will benefit from the application of AI in CADD.

Finally, the use of CADD tools requires a variety of expertise of researchers to perform all of the steps of the process, such as selecting and preparing targets and ligands, analyzing the results and having broad knowledge of computation, chemistry and biology. Thus, the researcher's background is important for the selection of new hits and to enrich high throughput experiments.

## Author Contributions

All authors of this review have made a great contribution to the work. All authors wrote the paper and approved the final version.

## Conflict of Interest

The authors declare that the research was conducted in the absence of any commercial or financial relationships that could be construed as a potential conflict of interest. The handling editor declared a past co-authorship with the author LA.

## Footnotes

^1^http://accelrys.com/products/collaborative-science/databases/bioactivity-databases/mddr.html
